# RELATION OF FEMUR FRACTURES LOCATION WITH CLINICAL OUTCOMES IN ELDERLY PATIENTS

**DOI:** 10.1590/1413-785220233101e239997

**Published:** 2023-04-17

**Authors:** Burak Celik, Ataman Kose, Abtullah Milcan, Akif Yarkac, Seyran Bozkurt Babus, Semra Erdogan

**Affiliations:** 1 Sanlıurfa Suruc State Hospital, Emergency Service, Sanlıurfa, Turkey.; 2 Mersin University, Faculty of Medicine, Department of Emergency Medicine, Mersin, Turkey.; 3 Mersin University, Faculty of Medicine, Department of Orthopedics and Traumatology, Mersin, Turkey.; 4 Mersin University, Faculty of Medicine, Department of Biostatistics and Medical Informatics, Mersin, Turkey.

**Keywords:** Femoral Fracture, Incidence, Aged, Mortality, Emergency Medical Services, Fratura Femoral, Incidência, Idoso, Mortalidade, Servicios Médicos de Urgencia

## Abstract

**Objectives:**

This study was designed to investigate the possible relationship between the anatomical location of the PFF (head-neck fractures) and the demographic features, comorbidities, and risk factors of elderly patients.

**Methods:**

233 patients aged 65 years and over, who were admitted to the emergency department with a diagnosis of proximal femur fracture were included in this study.

**Results:**

Most patients (59.6%) had a trochanteric fracture. The incidence of trochanteric fractures had a statistically significant positive correlation with age. Falls at ground level were found to be highly associated with trochanteric fractures (92,8%). At least one complication was observed in 57 (41,0%) cases and 31 (22,3%) died in one year, of the patients with trochanteric fractures. Comorbidity was not related to fracture location statistically. Fall ground level (p = 0.013), complication rate (73.7%; p <0.001), and Charlesen comorbidity index (p = 0.019) were statistically significantly associated with death. The logistic regression analysis of variables determined that only the quantity of comorbidities may be related to femoral neck fractures (p = 0.047).

**Conclusion:**

Female patients with trochanteric fractures were found to be older than male patients. Fall ground level, mortality, and complications were more frequently seen in patients with trochanteric fractures. ***Level of Evidence II, Retrospective study.***

## RESUMO

**Objetivos:**

Este estudo foi desenhado para investigar a possível relação entre a localização anatômica da PFP (fraturas de cabeça e pescoço) e as características demográficas, comorbidades e fatores de risco de pacientes idosos.

**Métodos:**

Foram incluídos 233 pacientes com idade igual ou superior a 65 anos, admitidos no pronto-socorro com diagnóstico de fratura do fêmur proximal.

**Resultados:**

A maioria dos pacientes (59,6%) apresentou fratura trocantérica. A incidência de fraturas trocanterianas apresentou correlação positiva estatisticamente significativa com a idade. Verificou-se que as quedas no nível do solo estão altamente associadas às fraturas trocantéricas (92,8%). Pelo menos uma complicação foi observada em 57 (41%) casos e 31 (22,3%) morreram em um ano, dos pacientes com fraturas trocantéricas. A comoborbidade não foi realizada estatisticamente com a localização da fratura. Queda do nível do solo (p = 0,013), taxa de complicações (73,7%; p < 0,001), índice de comorbidade charlesen (p = 0,019) foram estatisticamente significativamente associados à morte. Na análise de regressão logística das variáveis, determinou-se que apenas a quantidade de comorbidades pode estar relacionada às fraturas do colo do fêmur (p = 0,047).

**Conclusão:**

Pacientes do sexo feminino com fraturas trocantéricas foram consideradas mais velhas do que pacientes do sexo masculino. Queda no nível do solo, mortalidade e complicações foram observadas com mais frequência em pacientes com fraturas trocantéricas. ***Nível de evidência II, Estudo retrospectivo***.

## INTRODUCTION

Proximal femoral fractures (PFF) are a frequent cause of admission to the emergency department (ED), particularly in elderly patients and are associated with higher mortality, morbidity, and healthcare costs than any other osteoporotic fractures.^1,2^ About 250,000 hip fractures occur annually in the United States (US) and it is expected to increase over the coming years, due to the aging population. The mortality rate in patients with PFF in the US is 7% within a month and 24% within a year.^3^ PFFs are an important economical burden for healthcare. Medical treatment for injuries cost around 8.68 billion dollars.^4^

The known risk factors for PFF are; old age, female gender, sedentary lifestyle, alcohol and tobacco consumption, benzodiazepines, anticonvulsant drugs, cerebrovascular events, diabetes, osteoporosis, hyperthyroidism and some other chronic diseases.^5^ Besides that, some morbidities and mortality may develop after PFF. Therefore, it is important to assess comorbidities, potential risk factors, and monitor patients closely for prospective complications.^6,7^

Even a low-energy trauma (like a simple fall) may result in PFF. Approximately 30% of people aged 65 and over fall once a year.^8,9^ Many researchers have considered hip fractures as a single, homogeneous condition. Based on their anatomical locations, there are two main types of PFF: Trochanteric fractures and head-neck fractures. The potential risk factors affecting the type of fractures were investigated only in a few studies.^2,5^ Our study aims are to analyze the possible predisposing factors for the type of PFF and the prognoses of the patients. Several studies suggested that the type of fractures and their outcomes may be associated with some factors addressing the need for new studies.^2,5,10^ The type of PFF may affect mortality as well. Nevertheless, very few details were found on patients with proximal femur fractures who applied to the ED.

This study aimed to investigate a potential association between risk factors, comorbidities, socio-demographic and clinical features, and the type of PFF (trochanteric fractures and head-neck femur fractures) in patients presenting aged 65 years and over.

## MATERIALS AND METHODS

### Study Design

After the approval from the ethical board (dated 26.04.2018 and numbered 2018/188) patients aged 65 and over who were admitted to a University Hospital ED with a diagnosis of PFF between 01.01.2016-30.04.2018 were included in this study. As a result, 233 patients aged 65 and over who met the inclusion were included in the study. Our study was conducted by scanning the data of the patients according to the ICD-10 diagnostic codes via Nucleus, the hospital electronic information operating system, and confirmed with radiographs. All data were analyzed retrospectively. A descriptive cross-sectional study was conducted. Before the study, approval was received from the Clinical Research Ethics Committee dated 26.04.2018 and numbered 2018/188.

### Parameters of the Study

First, the location of PFF was classified as trochanteric or head/ neck. Intertrochanteric fractures, major and minor trochanteric fractures, and unspecified fractures in this region, were included in the group of trochanteric fractures. Intracapsular, femoral neck, femoral head, sub-capital, and unspecified fractures in this region, were recorded as femoral neck fractures.

Age, gender, mechanism of injury, comorbidities, Charlson Comorbidity Index (CCI), concomitant injuries, the American Society of Anesthesiologists (ASA) score, length of hospitalization, time to surgery, complications, causes of death were evaluated in these patients.

The patients were classified in three groups by their age as 65-74, 75-84, and more than 84. Comorbidities were assessed according to the CCI and classified as mild in the presence of 0-2 comorbidities, moderate into the presence of 3-4 comorbidity, and serious in the presence of 5 and more comorbidities. Time-to-surgery was reported in 24 hour-intervals for each patient. According to the ASA scores, ASA 1 was described as mild risk, ASA 2 as moderate risk, ASA 3 as high risk, and ASA 4 as very high risk. Deaths due to all causes occurring within 1 year were recorded.

Patients with incomplete data, under 65 years of age, patients without proximal femur fractures, were not included in the study.

### Statistical analysis

The Shapiro-Wilk test was used to determine if the continuous variables were normally distributed. Mann Whitney U test was used for differences between some parameters according to the location of the fracture. The Student t-test was used for the differences between the average ages. Mann Whitney U test was used for differences between continuous measurements according to primary and secondary causes of death. Average and standard deviation values are given for those with normal distribution in descriptive statistics. For those who do not have a normal distribution, minimum, maximum, median, 25-75% percentages are given. Pearson's chi-square and Likelihood ratio chi-square tests were used for categorical variables. Logistic regression analysis was applied in terms of some parameters according to the fracture type and survival status. Statistical significance was taken as p <0.05.

## RESULTS

Between the dates of 01.01.2016-30.04.2018, 233 patients over 65 years of age with PFF were admitted to ED, Trochanteric fracture was found in 59.7% (n = 139) of these patients, and neck fracture in 40.3% (n = 94) (Figure 1). 149 of the patients were women and 84 men.

The mean age of patients with trochanteric fracture (82,03±7,0) was higher than the patients with neck fracture (79.62±7.8) (p = 0.015). Majority of patients (%83,4) aged 75 years and over were diaognosed with trocanteric fracture (p = 0.049). (Table 1) The majority of PFFs were found to be fall ground level (n = 220, 94.4%) (p = 0.178). There was no accompanying injury in 91% (n = 212) of PFFs. 79% (n = 184) of patients were operated. HT was present in 45.9% (n = 107) of patients, DM in 35.2% (n = 82) of the patients whereas and total %87,5 of them had at least one comorbidity.

**Table 1:** Demographic and clinical features of the patients by fracture site.

Parameters	All patients (n=233)	Neck (n=94, 40.3%)	Trochanteric (n=139, 59.7%)	P
Age (Mean SD)	81,06 7,4	79,62 7,8	82,03 7,0	0,015
**Age groups**				**0,049**
65-74 age	50 (21.5)	27 (28.7)	23 (16,5)	
75-84 age	97 (41.6)	39 (41,5)	58 (41,7)	
≥85 age	86 (36.9)	28 (29,8)	58 (41,7)	
Gender, Women	149(% 63.9)	61 (% 64,9)	88 (63,3)	0,805
**Trauma mechanism**				**0,178**
Fall from height	4 (1.7)	0 (0,0)	4 (2,9)	
NVTA	7 (3.0)	3 (3,2)	4 (2,9)	
IVTA	2 (0.9)	0 (0,0)	2 (1,4)	
Fall ground level	220 (94.4)	91 (96,8)	129 (92,8)	
Accompanying injury	21 (9.0)	7 (7,4)	14 (10,1)	0,492
Mortality	38 (16.3)	7 (7,4)	31 (22,3)	0,003
Complication	81 (34.8)	24 (25,5)	57(41,0)	0,015
Fracture side, Left	130 (55.8)	53 (56,4)	77 (55,4)	0,882
Fracture side, Right	103(44.2)	41 (43,6)	62 (44,6)	
Surgical condition	184 (79.0)	79 (84,0)	105 (75,5)	0,118
**ASA Score**				**0,670**
Moderate	39 (20.4)	19 (23,5)	20 (18,2)	
High	83 (43.5)	34 (42,0)	49(44,5)	
Highest	69 (36.1)	28 (34,6)	41 (37,3)	
**Length of stay**				**0,696**
<7 days	161 (69.0)	64 (80,0)	97 (82,2)	
>7 days	37 (15.8)	16 (20,0)	21 (17,8)	
Mild comorbidity index	135 (57.9)	52 (55,3)	83 (59,7)	0,596
Moderate comorbidity index	54 (23.1)	25 (26,6)	29 (20,9)	0,393
Severe comorbidity index	15 (6.4)	7 (7,4)	8 (5,8)	0,829
Comorbidity	204 (87.5)	84 (89,4)	120 (86,3)	0,492
**Comorbidities**				
DM	82 (35,2)	33 (35,1)	49 (35,3)	0,982
HT	107 (45,9)	47 (50,0)	60 (43,2)	0,304
Alzheimer/demans	52 (22,3)	23 (24,5)	29 (20,9)	0,517
Parkinson's disease	14 (6,0)	8 (8,5)	6 (4,3)	0,186
Cerebrovascular disease	15 (6,4)	6 (6,4)	9 (6,5)	0,978
Malignity	26 (11,2)	11 (11,7)	15 (10,8)	0,829
Congestive heart failure	29 (12,4)	10 (10,6)	19 (13,7)	0,492
Kidney failure	42 (18,0)	18 (19,1)	24 (17,3)	0,714
Coronary arter disease	26 (11,2)	14 (14,9)	12 (8,6)	0,136
Asthma/COPD	21 (9,0)	6 (6,4)	15 (10,8)	0,249
Chronic liver disease	2 (0,9)	2 (2,1)	0 (0,0)	0,056
Rheumatological diseases	8 (3,4)	4 (4,3)	4 (2,9)	0,575
Time to surgery (days)*	2 [2-4]	2 [1-4]	3 [2-4]	0,075
Number of comorbidities*	2 [1-3]	2 [1-3]	2 [1-3]	0,236
Length of stay (days)*	5 [4-7]	5 [4-7]	5 [4-7]	0,908

Data are expressed as mean ± SD, or number (percentage) or

* Median [% 25-75 percentiles]. NVTA: Non-vehicle traffic accident, IVTA: In-vehicle traffic accident, DM: Diabetes Mellitus, HT: Hypertension, COPD: Chronic obstructive pulmonary disease.

No statistically significant difference was found between trauma mechanism, accompanying injury, surgical condition, fracture location, ASA score, length of stay, comorbid index, comorbidity status, number of comorbidities, time to surgery, and fracture side (p> 0.05). Complication rate was 34.8% (n =81). Patients with trochanteric fractures had statistically significantly more complication rates (% 41) than patients with neck fractures (% 25,5) (p=0.015). (Table 1) It was determined that 16.3% of the patients with PFF died within 1 a year The mortality rate after one year of follow-up was significantly higher in patients with trochanteric fractures than neck fractures, which were %22,3 and %7,4 respectively (p=0.003). (Table 1)

In the logistic regression analysis of variables affecting the fracture type, only the number of comorbid was determined to be effective on the fracture type. Accordingly, it was determined that the comorbid number of trochanteric fractures is lower than femoral neck fractures (p = 0.047). (Table 2)

**Table 2:** Logistic regression analysis of variables that affect fracture type.

Variables	B	Wald	OR [95% CI]	P
Age	0,040	3,17	1,041 [0,996-1,089]	0,075
Trauma		0,382		0,933
Fall from height vs fall ground level	21,371	0,000	-	0,999
NVTA vs Fall ground level	-0,592	0,382	0,553 [0,085-3,616]	0,510
IVTA vs Fall ground level	20,196	0,000	-	0,999
In life vs Death	0,847	2,674	2,333 [0,845-6,443]	0,102
Complication Yes vs No	0,499	2,140	1,647 [0,844-3,215]	0,143
Number of comorbidities	-0,250	3,931	0,778 [0,608-0,997]	0,047
Time to surgery	0,119	1,400	1,126 [0,925-1,371]	0,237

NVTA: Non-vehicle traffic accident, IVTA: In-vehicle traffic accident.

There was no statistically significant correlation between the mortality and the gender, age, side of the fracture, time-to-surgery of the patient. (Table 3) However, It has been found that mortality is associated with fall ground level (p = 0.013), complication development (p <0.001), comorbid index (p = 0.015), high ASA score (p = 0.001), length of stay (<7 days) (p = 0.017). (Table 3) The difference between the number of comorbidities was found to be significant in patients who survived after 1 year (p = 0.008). Although the median values are equal, the mean rank values of those who died (mean rank = 142.75) were significantly higher than those who lived (Mean rank = 111.98).

**Table 3:** Demographic and clinical relationship of patients according to their survival status.

Variables	Dead 38 (16.3% )	Alive 195 (83,7% )	P
Age(Mean SD)	82.1 7.1	80.9 7.5	0,338
**Age groups**			**0,595**
65-74 ages	6 (15,8)	44 (22,6)	
75-84 ages	18 (47,4)	79 (40,5)	
≥85 ages	14 (36,8)	72 (36,9)	
Gender, Women	23 (60,5)	126 (64,6)	0,631
**Trauma mechanism**			**0,013**
Fall from height	2 (5,3)	2 (1,0)	
NVTA	2 (5,3)	5 (2,6)	
IVTA	2 (5,3)	0 (0,0)	
Fall ground level	32 (84,2)	188 (96,4)	
Complication	28 (73,7)	53 (27,2)	<0,001
Comorbidity	35 (92,1)	169 (86,7)	0,509
Mild comorbidity index	17 (44,7)	118 (60,5)	0,015
Moderate comorbidity index	11 (28,9)	43 (22,1)	
Severe comorbidity index	7 (18,4)	8 (4,1)	
Fracture side, Left	18 (47,4)	112 (57,4)	0,253
Fracture side, Right	20 (52,6)	83 (42,6)	
Surgical condition	28 (73,7)	156 (80,0)	0,382
Time to surgery	3 [2-4]	2 [1-4]	0.099
ASA score Moderate	1 (3,2)	38 (23,8)	0,001
ASA score High	10 (32,3)	73 (45,6)	
ASA score Highest	20 (64,5)	49 (30,6)	
Length of stay <7 days	25 (67,6)	136 (84,5)	0,017
Length of stay >7 days	12 (32,4)	25 (15,5)	
Number of comorbidities *	2 [1-3,25	2 [1-2]	0,008
Length of stay *	6 [4-9]	5 [4-7]	0,112

Data are expressed as mean ± SD, or number (percentage) or

* Median [% 25-75 percentiles]. NVTA: Non-vehicle traffic accident, IVTA: In-vehicle traffic accident.

## DISCUSSION

Several studies have been conducted on PFFs concerning diagnosis, treatment, and survival according to the fracture location, and various results have been achieved. In the study of Endo et al. in PFFs over 65 years of age 50.8% were femoral neck fractures and 48.2% intertrochanteric fractures.^11^ In our study, neck fractures were detected in 40.3% of patients and trochanteric fractures in 59.7%. The potential association between the age and type of fracture was also studied. Diaz et al.^5^ reported no association between these variables, in contrast, some studies showed trochanteric fractures were observed more often in patients with an increased age which was also further supported in our study.^2,10^ We don't know yet, whether the level of osteoporosis or another possible alteration in bone morphology with increasing age influences the fracture type. According to our opinion, this is an obscure field that should be investigated. In our study, we were not able to identify any other relevant variables that may affect the type of fracture.

The relation between the type of hip fracture and mortality rate is controversial. In many mortality studies, patients with trochanteric fractures were found to have a higher mortality rate than those with femoral neck fractures,^2,12^ whereas Kim et al. reported that cervical fracture had a higher risk for mortality than trochanteric fracture^13^ and some studies did not show an association.^14,15^ We found that the mortality rate after trochanteric fracture to be higher. However, this may be associated with the higher mean age of the trochanteric fracture group in our study. Similar mortality rates in patients older than 85 years old may be accepted as a supporting finding to our conjecture. Increased risk of mortality is expected with the increasing age in patients older than 65 years with hip fractures.^16^ Nevertheless, we found that the highest mortality rate of 47.4%. was in patients between 75 and 84 years old. We think that this might be associated with a higher rate of comorbidities in this group. In a mortality study of Kesmezacar et al. in patients over 65; 57.9% of 76 male patients and 41.9% of 172 female patients died. The overall mortality rates were significantly higher in men than in women.^15^ In our study, no gender differences were found in the mortality rates.

It has been found that 82% of patients with femoral fractures have an important medical condition that contributes to or complicates the fracture.^6^ In the study in which Diaz et al. examined the risk factors for trochanteric and femoral neck fractures, the number of comorbidities was between 5-9, 35% patients with neck fractures, and 47.1% patients with trochanteric fractures were identified. This was found statistically significant.^5^ In a study by Fox et al. on 923 patients with proximal femur fractures over 65 years of age; 4 or more comorbid diseases were detected in 82.3% of intertrochanteric fractures and 76.1% of neck fractures.^2^ In our study, 59.7% of patients with trochanteric fractures had 1-2 and 55.3% of patients with neck fractures had 1-2 comorbid diseases. CCI has been applied in outcome studies on elderly patients with hip fractures, and a meta-analysis revealed that the zero scores have a 41% lower risk of death compared to those with one or more CCIs.^17^ In our study, consistent with the literature, the probability of mortality was higher in patients with high CCI score.

There are contradictory reports regarding the association of the mechanism of trauma and the localization of the fracture.^8,18^ Our study showed no association between these variables. To classify most of the patients in a group as falls may be misleading since the different acting forces in the fracture region are not considered. In the study of mortality in the first year after the proximal femoral fracture in elderly patients, mortality increased as the ASA score increased.^19^ Our study is compatible with the literature, and it has been determined that mortality increases with the increase in the ASA score.

The ideal time for surgical repair of hip fractures is controversial. Early surgical treatment is associated with independent return to life, shorter hospital stays, and 1-year survival rates. These studies are related to general hip fractures.^20,21^ Nevertheless, there is insufficient data regarding the location of the fracture and its timing of the operation. In a study related to the time of surgery according to the fracture location, the average delay to surgery in patients over 65 years of age was 8.7 days for trochanteric femur fractures and 11.3 days for femoral neck fractures.^15^ Different results have been reported in studies conducted on the effect of time to surgery on 1-year mortality in hip fractures.^22,23^ In our study, when trochanteric and head/neck fractures were examined within themselves, the time until surgery did not affect on mortality. However, we think that this can be caused by the operation of trochanteric fractures in an average of 3 days and neck fractures in a short time such as 2 days. In studies involving hip fractures operated later in the literature, we could not find any data showing the relationship between different types of fractures and mortality.

A previous study reported longer hospitalization duration for patients with trochanteric fractures (19.7 days) than femoral neck fractures (17.5 days).^2^ Approximately 80% of patients with fractures were hospitalized for up to 7 days in our study. Umarji et al. reported that if patients with PFFs stay in the hospital for longer than 8 days, this would not benefit the patient since most patients get a nosocomial infection after 8 days^24^ which was also consistent with our study. It is also highly possible that the preoperative conditions of these patients could be the reason for the longer hospitalization that eventually leads to a higher mortality rate.

The incidence of postoperative complications after hip fractures was found to be 20%.^23^ However, the potential role of the fracture location on complications was not investigated before. In our study, 34.8% of cases developed complications after a fracture. In our study, 41% of patients developed complications after trochanteric fracture, which was considered significant. Complications were found in 73.7% of patients who died. Higher mean age and comorbidity rates in the trochanteric fracture group may be the reason for this.

Our study had several limitations. One is the retrospective nature of the study and its being single-centered. Another limitation is the possible lack of patient data through file scanning. Some variables such as Alcoholism, hyperthyroidism, or hypovitaminosis D, body mass index, and geometry of the treated hip could not be studied due to insufficient data. Further prospective studies with larger groups are needed.

## CONCLUSION

In conclusion; the majority of the patients with PFFs admitted to the ED are trochanteric fractures. Most of these patients are female and the frequency of fractures increases as the average age increases. In trochanteric fractures, fall ground level, mortality, complications, and surgical intervention are more common. There is no difference in the presence of comorbidity between both types of fractures, but in logistic regression analysis, only the number of comorbidities is effective on the fracture type. In general, fall ground level, development of complications, comorbid index, increased comorbid count, high ASA score, and length of stay are the effective factors found in patients who died due to PFF**.** Patients who develop complications have a higher risk of death. We believe that our findings can guide healthcare professionals and new research in terms of approach to patients applying to the ED with a proximal femur fracture.
